# Supplementary material for: Acquiring new memories in neocortex of hippocampal-lesioned mice
Source: Nat Commun. 2022 Mar 24;13:1601. doi: 10.1038/s41467-022-29208-5 (PMC8948206; doi:10.1038/s41467-022-29208-5)
Supplement: Supplementary file 1 — Supplementary Information [file 41467_2022_29208_MOESM1_ESM.pdf]

Acquiring new memories in neocortex of hippocampal-lesioned mice

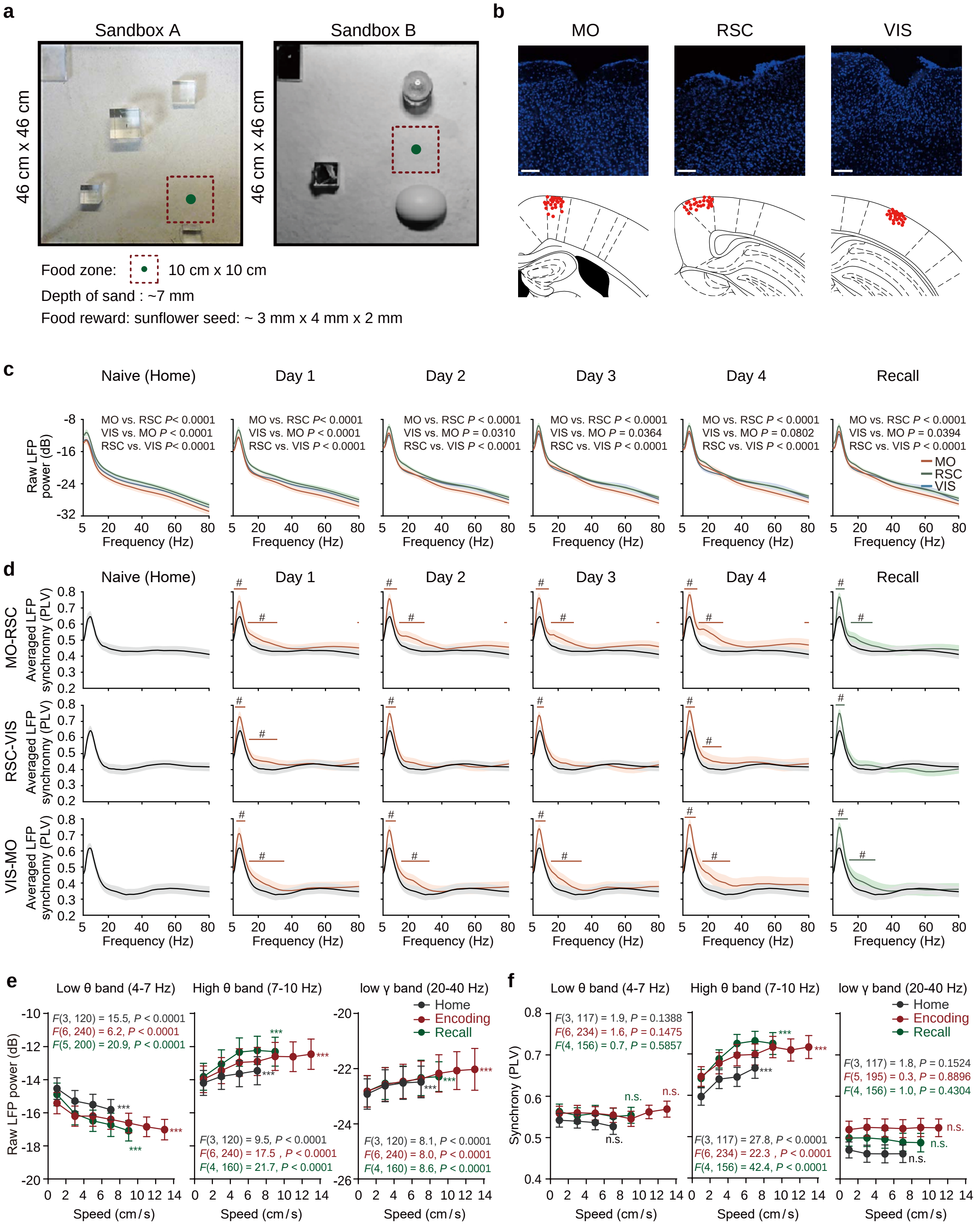

Supplementary Figure 1

Supplementary Fig. 1. Cortical gamma synchronization is not affected by locomotion and is widespread among cortical region pairs.

a, Images of sandboxA and sandboxB for the spatial memory task, size information is noted under the images. Green dot, location of the food reward; Dash square, the definition of the food zone. Landmarks and the food location were different in two boxes. SandboxA had white walls and sandboxB had black walls. There was no stripe or any other sign was on the wall to provide spatial information. Five transparent glass blocks were placed in the sandboxA as the spatial landmark, while four non-transparent objects were placed in the sandboxB as the landmark. Mice start exploring from the starting point in all trials. **b,** Electrodes were targeted to the cortical superficial layers. Top, sample images of three regions show the damage by the electrode. Bottom, targeted point of all mice. Although targeted points vary across animals, they were mainly located at the superficial layers. Animals with off-target electrodes were excluded from the dataset. $N_{MO} = 26$, $N_{RSC} = 26$, $N_{VIS} = 26$ from 28 mice. Scale bar, 100μm. **c,** Evolution of raw LFP powers. Significance was assessed by two-way ANOVA followed by false discovery rate (FDR) corrected multiple comparisons between regions, corresponding P values are noted on the figure, $N_{MO} = 26$, $N_{RSC} = 26$, $N_{VIS} = 26$ from 28 mice). **d,** Evolution of synchrony from three brain regions pairs. Grayline, homecage synchrony for comparison (Two-way ANOVA, #$q < 0.05$, FDR corrected, significant frequency range is noted on the graph, $N_{MO\text{-}RSC} = 24$, $N_{RSC\text{-}VIS} = 24$, $N_{MO\text{-}VIS} = 24$ from 28 mice). **e,** Curves of cortical LFP power with respect to the moving speed of the animal (n = 41 electrode). **f,** Curves of cortical LFP synchrony with respect to the moving speed of the animal (n = 40 electrode pairs). Low theta and low gamma synchronization were not affected by the locomotion of mice. Statistical significances were assessed by one-way ANOVA. Statistical parameters are noted on the graph in the corresponding color. Error bars and shadow of line show S.E.M. n.s., no significance. ***$P < 0.001$.

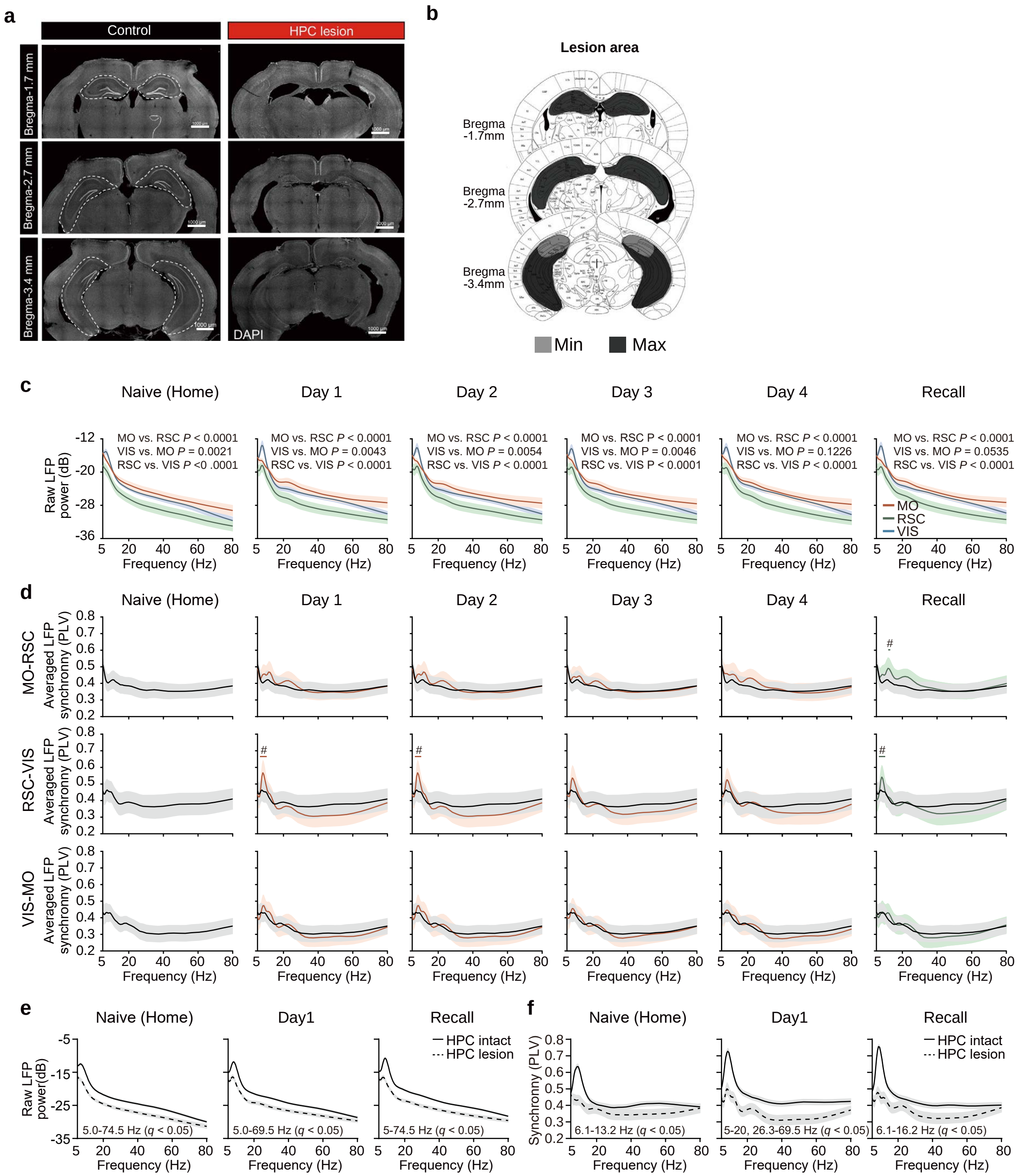

Supplementary Figure 2

Supplementary Fig. 2. Hippocampus regulates cortical LFP power and synchronization.

a-b, Bilateral NMDA injection causes severe lesions in both dorsal and ventral hippocampus. **a,** Examples of coronal sections of HPC-lesioned mice and control mice (15 mice brain were sectioned, represnetive images here are from one of them). Dash line regions, HPC areas. Although there were still remnant tissues in the hippocampus, cells and structures inside were damaged, scale bar, 1000μm. **b,** The max and min lesion areas in all HPC-lesioned mice (N = 15 mice). **c,** Evolution of raw LFP powers of HPC-lesioned mice. Significance was assessed by two-way ANOVA followed by false discovery rate (FDR) corrected multiple comparisons between regions, corresponding P values were noted on the figure, N_{MO} = 11, N_{RSC} = 12, N_{VIS} = 12 from 12 mice). **d,** Evolution of synchrony from three brain regions pairs of HPC-lesioned mice. Grayline, homecage synchrony for comparison. (Two-way ANOVA, #$q < 0.05$, FDR corrected, significantly changed frequencies were noted on the graph, $N_{MO\text{-}RSC}$ = 11, $N_{RSC\text{-}VIS}$ = 12, $N_{MO\text{-}VIS}$ = 11, from 12 mice). **e,** Comparison of overall power between HPC intact mice and HPC-lesioned mice. (78 electrodes from 28 HPC intact mice; N_{MO} = 26, N_{RSC} = 26, N_{VIS} = 26; 35 electrodes from 12 HPC-lesioned mice , N_{MO} = 11, N_{RSC} = 12, N_{VIS} = 11; $q < 0.05$. FDR corrected, the significant frequency range is noted on the graph). **f,** Comparison of overall synchrony between HPC-lesioned mice and HPC intact mice. (72 electrode pairs from 28 HPC-intact mice. $N_{MO\text{-}RSC}$ = 24, $N_{RSC\text{-}VIS}$ = 24, $N_{MO\text{-}VIS}$ = 24; 34 electrode pairs from 12 HPC-lesioned mice. $N_{MO\text{-}RSC}$ = 11, $N_{RSC\text{-}VIS}$ = 12, $N_{MO\text{-}VIS}$ = 11, #$q < 0.05$, FDR corrected, Significant frequency range is noted on the graph).

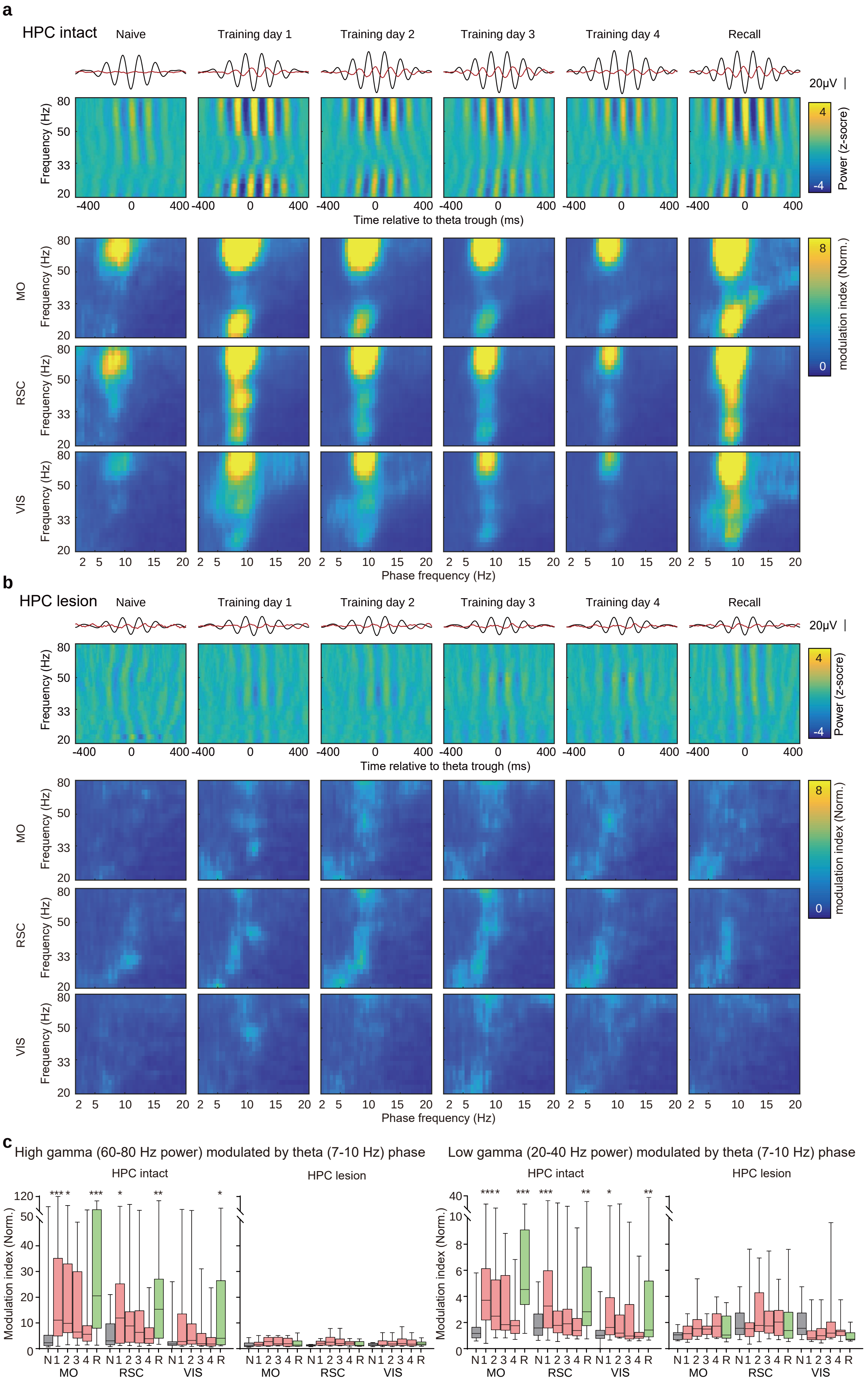

Supplementary Figure 3

Supplementary Fig. 3. Hippocampal-dependent coupling between cortical gamma power and theta phase is elevated during memory encoding and recall

a-b, The power-phase coupling of 3 cortical areas in learning stages. **a,** Results of HPC intact mice (Electrode number: N_{MO} = 26, N_{RSC} = 26, N_{VIS} = 26, from 28 mice). **b,** Results of HPC lesion mice (Electrode number: N_{MO} = 11, N_{RSC} = 12, N_{VIS} = 12 from 12 mice). Top, averaged theta wave (black) and 30Hz power (red). Middle, averaged phase(θ)-power(γ) spectrogram from all regions. Bottom, phase-power modulation index comodulograms of all regions. Modulation indexes were normalized by the element-wise division of the raw comodulograms by surrogated control. **c**, Quantification of modulation indexes in a and b. Significance measured by one-way ANOVA, *P < 0.05, **P < 0.01, **P < 0.001, n.s., not significant. For all box plots, whiskers show min and max, box shows 25th, median and 75th percentile.

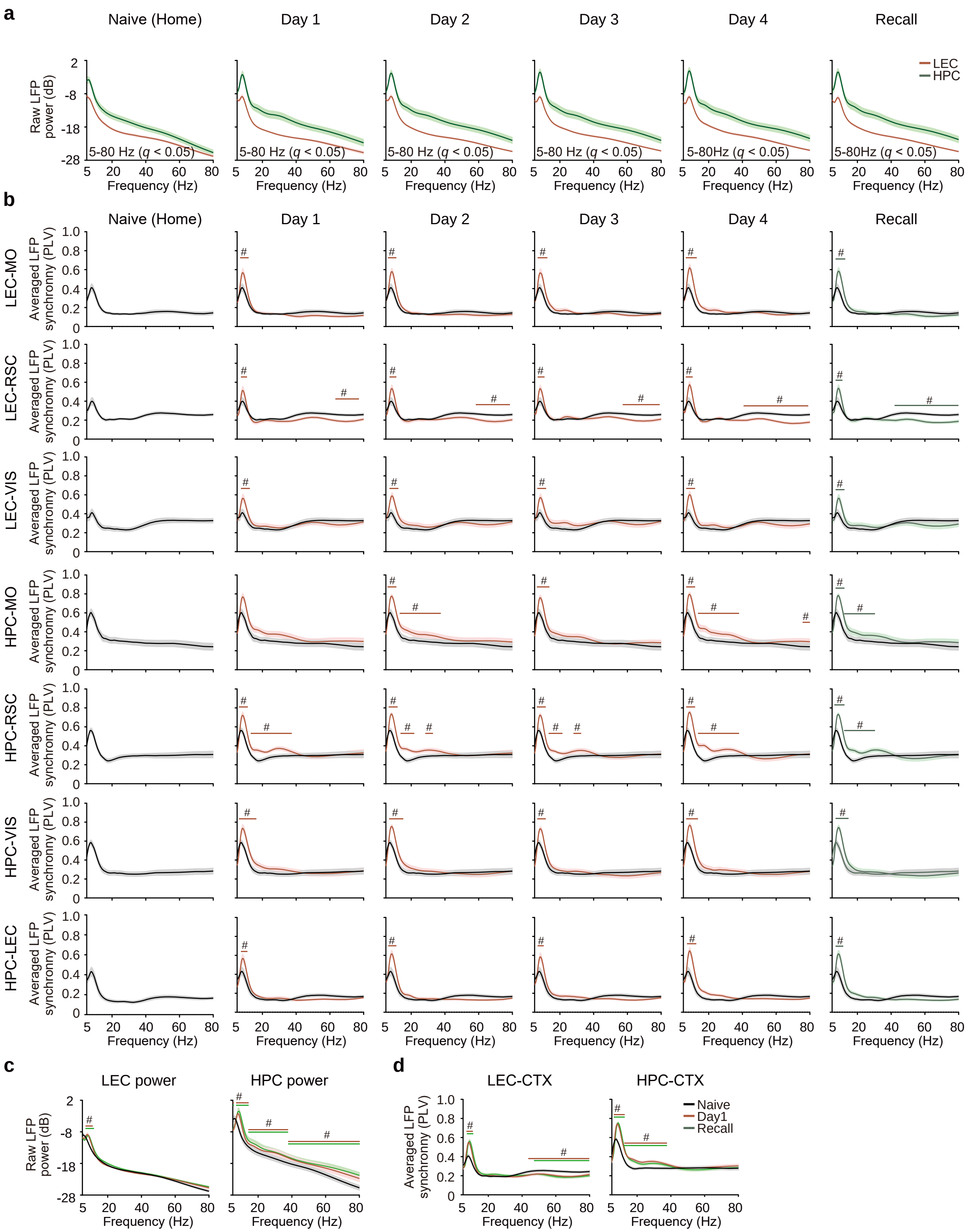

Supplementary Figure 4

Supplementary Fig. 4. The evolution of the power of LEC/HPC(CA1) and synchrony between HPC/LEC and various cortices.

a, The evolution of the LFP powers of LEC and HPC (N_{LEC} = 18, N_{HPC} = 17). **b,** The evolution of synchronies between LEC/HPC and cortices. N_{MO-RSC} = 19, $N_{RSC-VIS}$ = 19, N_{VIS-MO} = 19, N_{LEC-MO} = 18, $N_{LEC-RSC}$ = 18, $N_{LEC-VIS}$ = 18, N_{HPC-MO} = 17, $N_{HPC-RSC}$ = 17, $N_{HPC-VIS}$ = 17, $N_{HPC-LEC}$ = 16, from 19 mice. **c,** Encoding (day1) and recall trials showed significant theta power elevation in LEC, significant theta and gamma power elevation in HPC. **d,** Averaged synchronies between LEC/HPC and cortical regions. All significance was assessed by two-way ANOVA followed by false discovery rate (FDR) corrected multiple comparisons at each frequency comparing with data of homecage trail. Significant frequency range ($q < 0.05$) is noted on the graph. #*q* < 0.05, Shadow of line plot shows S.E.M.

a

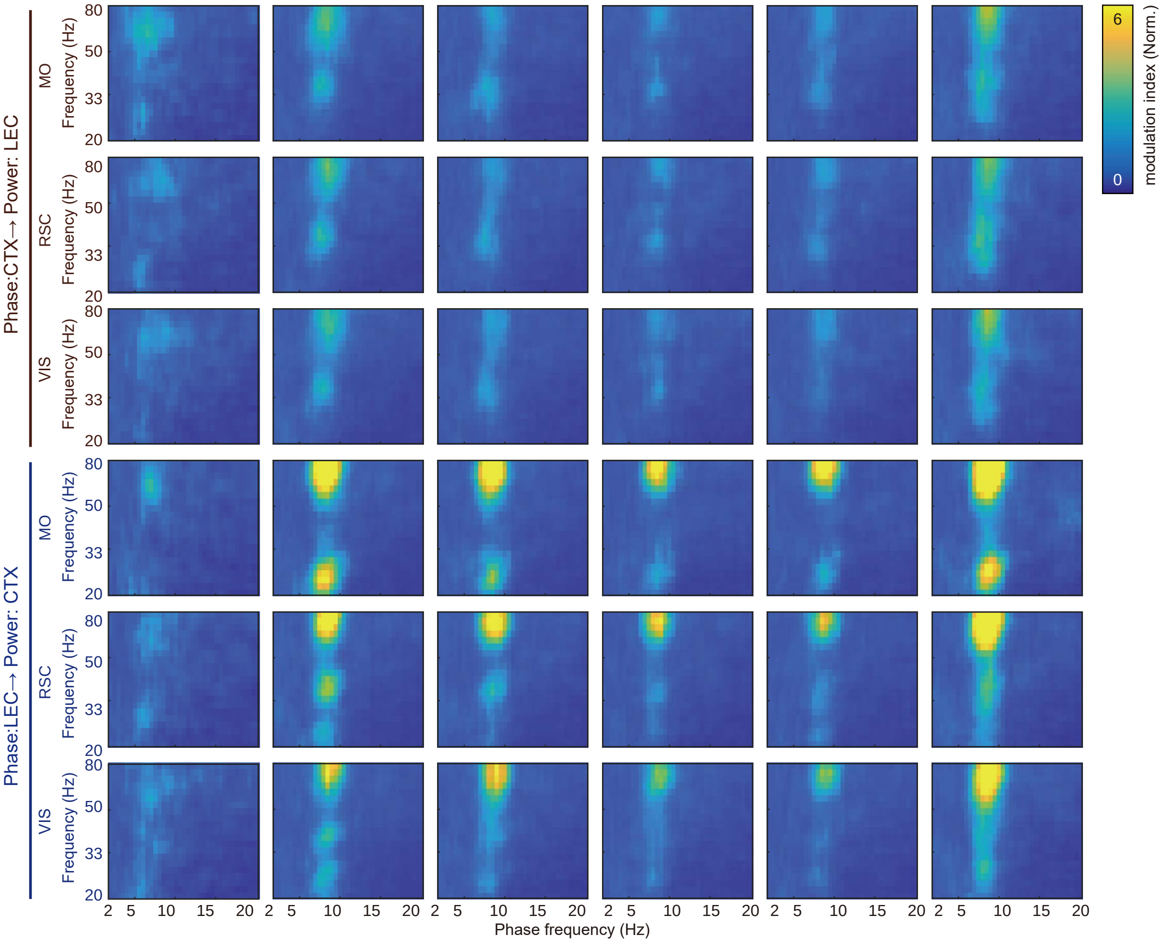

b

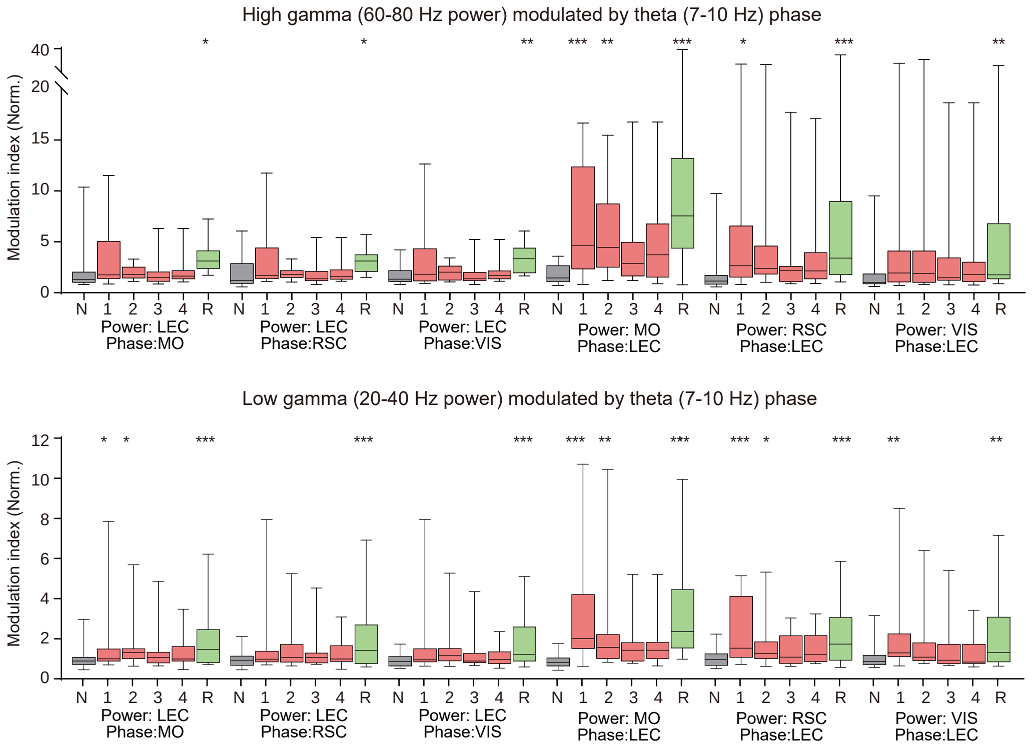

Supplementary Figure 5

Supplementary Fig. 5. Cortical gamma power is modulated by theta wave of LEC.

a, Phase-power modulation index comodulograms between cortices and LEC (Averaged from all pairs, N = 18). The modulation indexes between cortical gamma and LEC theta were stronger than the indexes between LEC gamma and cortical theta, indicating cortical gamma was modulated by LEC theta but not vice versa. **b,** Quantification of modulation index in a. Significance measured by one-way ANOVA, *P < 0.05, **P < 0.01, n.s., not significant. For all box plots, whiskers show min and max, box shows 25th, median and 75th percentile.

a

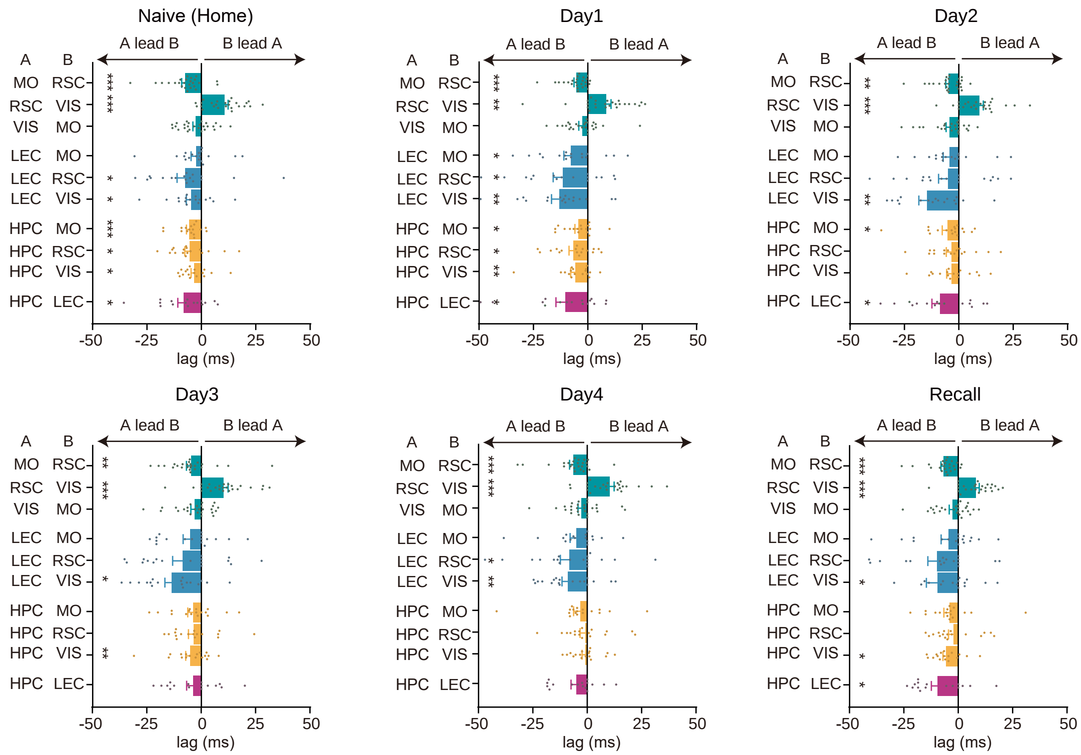

Supplementary Fig. 6. HPC theta leads to LEC theta and LEC theta leads cortical theta.

a, Theta wave amplitude based cross-correlation analysis of all learning stages and all pairs between regions. Statical significances were assessed by Wilcoxon signed-rank test, comparing to 0. $N_{MO\text{-}RSC}$ = 19, $N_{RSC\text{-}VIS}$ = 19, $N_{VIS\text{-}MO}$ = 19, $N_{LEC\text{-}MO}$ = 18, $N_{LEC\text{-}RSC}$ = 18, $N_{LEC\text{-}VIS}$ = 18, $N_{HPC\text{-}MO}$ = 17, $N_{HPC\text{-}RSC}$ = 17, $N_{HPC\text{-}VIS}$ = 17, $N_{HPC\text{-}LEC}$ = 16 from 19 mice. *P < 0.05, **P < 0.01, ***P < 0.001

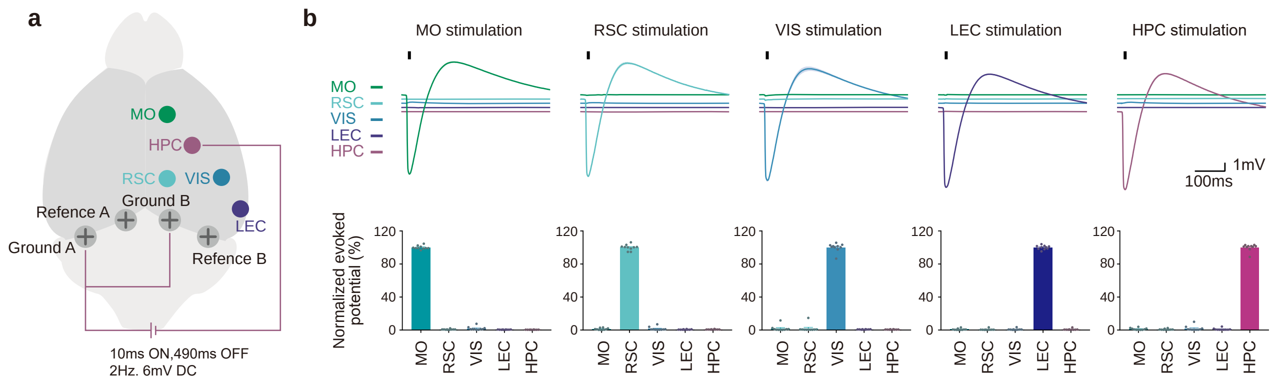

Supplementary Fig. 7. Volume conduction has little effect on multi-regional LFP recording.
a, Experimental diagram. One of five regions was electrically stimulated and the effect of volume conduction was quantified in other brain regions during mice awake in homecage. Stimulation voltage: 6V, DC, 10ms, 2Hz. Screws were implanted upon the transverse sinus. We chose the transverse sinus for the reference screw because the position below is less neuron and more vascular tissue. **b,** Top, averaged raw LFP traces in 5 brain regions with one of them was stimulated. Bottom, quantification of normalized evoked potentials in the upper panel. 50 electrodes from 10 mice.

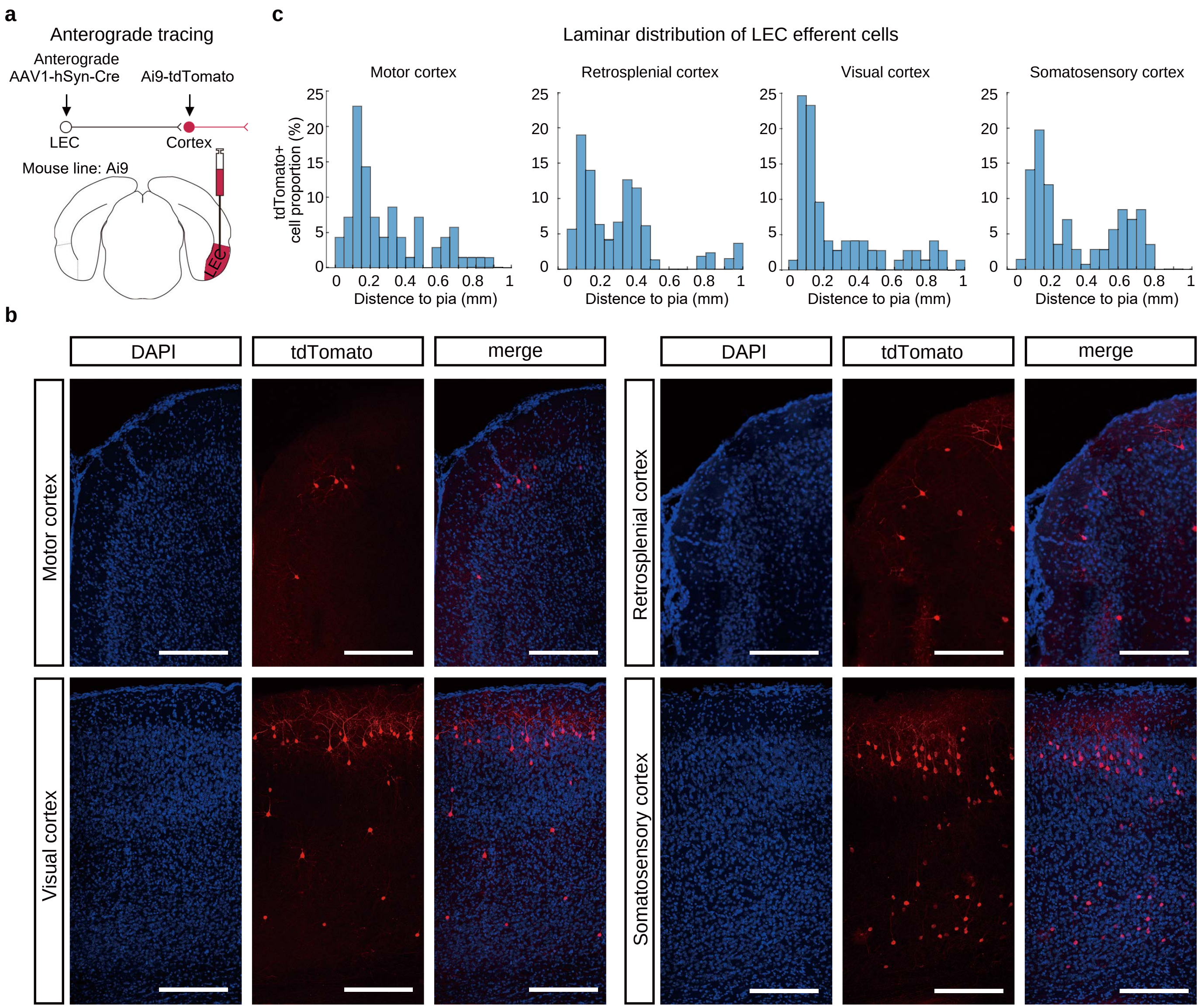

Supplementary Fig. 8. LEC projections mainly target to cortical layer 2/3 neurons.
a, Scheme of anterograde tracing of LEC efferent neurons infected by AAV1 in Ai9-tdTomato reporter mice. **b,** Example images of LEC downstream neurons in various cortical areas. Scale bar: 200μm. **c,** Quantification of laminar distribution of LEC efferent neurons from all regions (Mice: n = 3, Neuron number: N_{MO}= 70, N_{RSC} = 64, N_{VIS} = 102, N_{SS}= 70, in total 309 cells.

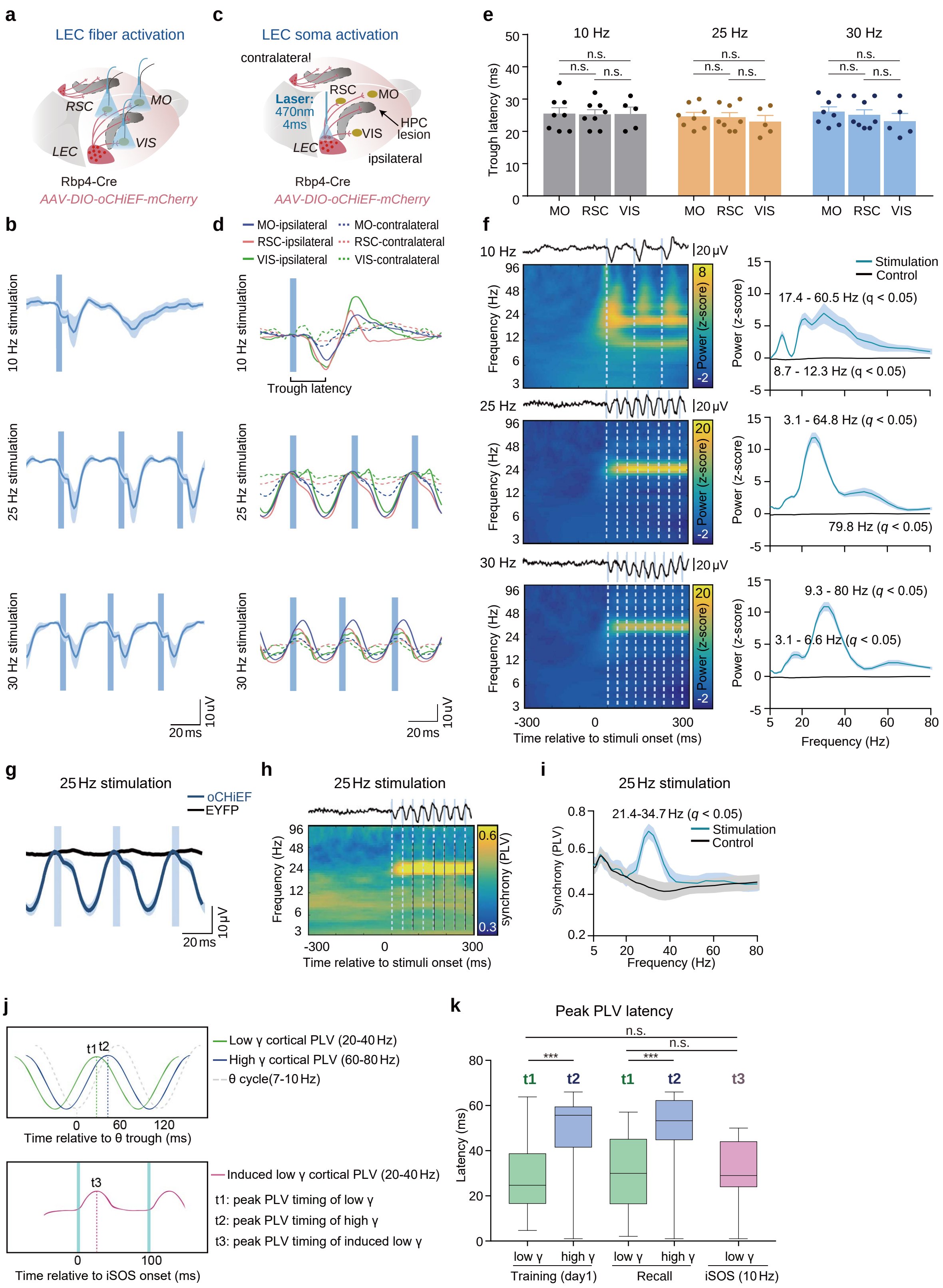

Supplementary Figure 9

Supplementary Fig. 9. LEC neuronal activation leads to synchronization among brain regions, similar to endogenous theta-modulated PLV rhythmicity.

a-b, Activating LEC cortical fibers induce synchronized cortical potentials. **a,** Multi-region recording, and laser stimulation of LEC cortical fibers simultaneously through optrodes in HPC-lesioned mice in homecage. Light pulse, 4 ms. **b,** Averaged induced potentials of different regions (electrode: n = 8). **c,** Experimental diagram for multi-region recording during activation of LEC neurons in HPC-lesioned mice (same as Fig. 2l). Golden circles, recording sites. Dash line region, lesioned hippocampus. Light pulse, 4 ms. **d-e**, LEC neurons activation caused zero-phase lag synchronization among cortical regions. **d,** Averaged induced potentials of different regions, including ipsilateral regions and contralateral regions. Trough latency refer to the latency from pulse onset to trough of the potential (electrode numbers: ipsilateral: MO = 8, RSC = 8, VIS = 5; contralateral: MO = 6, RSC = 6, VIS = 3). **e,** Quantifications of trough latency of three ipsilateral brain regions (electrode numbers for all kinds of stimulation: MO = 8, RSC = 8, VIS = 5; 10Hz stimulation: RSC vs. MO vs. VIS, ANOVA, $F(2, 18) = 0.0056$, $P = 0.9944$. 25Hz stimulation: RSC vs. MO vs. VIS, ANOVA, $F(2, 18) = 0.28$, $P = 0.7577$. 30Hz stimulation: RSC vs. MO vs. VIS, ANOVA, $F(2, 18) = 0.64$, $P = 0.5365$). **f,** Power spectrogram and quantification under 10Hz, 25Hz, 30Hz LEC neuronal activation. Left, power spectrograms. Right, corresponding quantifications of LFP powers before and after stimulation onset. Control, averaged power before onset; Stimulation, averaged power at 10-50 ms. ($q < 0.05$, FDR corrected. Electrode numbers for all stimulations: n = 21, significantly changed frequencies were noted on the graph)**. g-i**, Besides 10Hz and 30Hz activations mentioned in the main text, activating LEC neurons with 25Hz stimulation also induced cortical synchronization on corresponding frequency. **g,** Evoked cortical potentials under 25Hz LEC activation. The blue bar indicates the light pulse (n = 21 electrodes, averaged trace of RSC, MO, and VIS). **h,** PLV spectrogram of 25Hz LEC activation (average of 16 electrode pairs). **i,** Quantification of cortical synchrony before and after stimulation onset. Control, averaged PLV before onset; Stimulation, averaged PLV at 10-300 ms. ($q < 0.05$, FDR corrected, 16 electrode pairs, significantly changed frequency ranges are noted on the graph). **j-k**, iSOS produced PLV rhythmicity that was similar to endogenous theta-modulated PLV rhythmicity. **j,** Illustrations of peak latency of low gamma and high gamma PLV, relative to theta trough (top) and LEC neuron stimulation onset (bottom). **k,** Comparison of these peak PLV latency. (In training trials: latency from all 72 PLV pair; low gamma vs. high gamma, two-sided paired *t*-test, $t(71) = 8.1$, $P < 0.0001$; In recall trials: latency from same 72 PLV pair; low gamma vs. high gamma, two-sided paired *t*-test, $t(71) = 6.2$, $P < 0.0001$; iSOS stimulation: low gamma latency from same 16 PLV pair; Training low gamma vs. recall low gamma vs. iSOS low gamma, one-way ANOVA, $F(2, 157) = 0.66$, $P = 0.5173$, no significance is detected). Error bars show mean ± S.E.M. n.s., no significance. Shadow of line plot shows S.E.M.

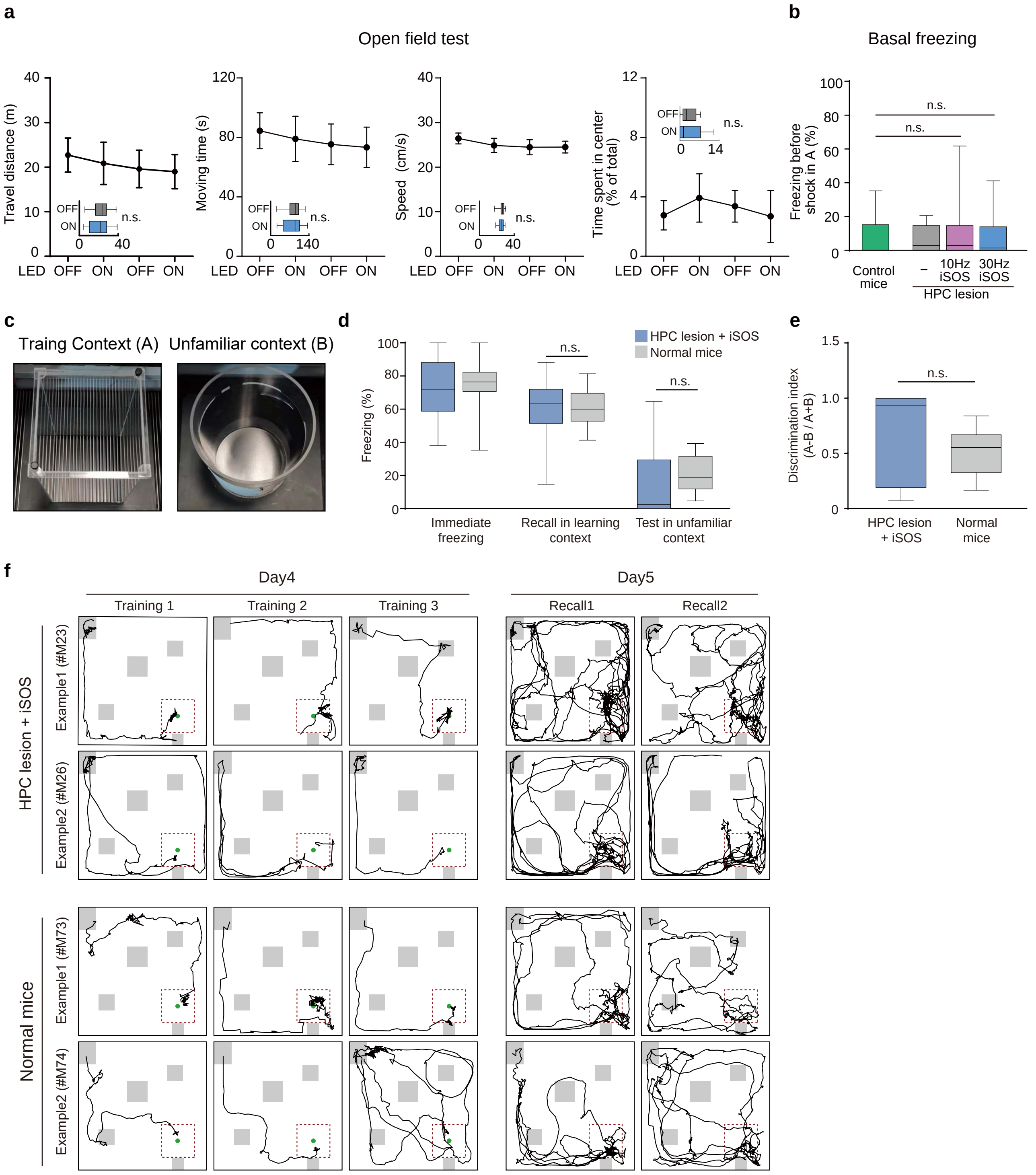

Supplementary Figure 10

Supplementary Fig. 10. Activation of cortical LEC fibers does not affect locomotor activities, anxiety level, nor changing basal freezing level, and shows the same memory fidelity as normal mice.

a, Quantifications of four parameters of open field test (OFF-ON-OFF-ON, 3 mins for each block, n = 13), including travel distance (same as Fig.3c, moving time (two-sided paired *t*-test, $t(12) = 1.6$, $P = 0.1382$), moving speed (two-sided paired *t*-test, $t(12) = 0.76$, $P = 0.4626$) and time spent in center (two-sided paired *t*-test, $t(12) = 0.22$, $P = 0.8282$). **b,** Box plot of basal freezing levels before contextual fear conditioning (Mice number, Control = 15, HPC lesion = 11, $iSOS_{10Hz} = 8$, $iSOS_{30Hz} = 8$, ANOVA, $F(3, 38) = 0.1625$, $P = 0.9209$). Whiskers show min and max, box shows 25th, median and 75th percentile. **c-e**, Fear memory rescued mice show the same memory fidelity as normal mice. **c**, Images for two boxes. **d**, Part of mice in fig. 3a-e additionally underwent a unfamiliar context test. Mice were trained with protocol as shown in Fig. 3a-b, one dot represents data of one mouse. $N_{Normal\ mice} = 11$, $N_{HPC\ +\ iSOS} = 14$ (8 with 10Hz iSOS, 6 with 30Hz iSOS). Two-way ANOVA, Group factor, $F(1,23) = 0.1678$, $P = 0.6859$. **e**, Quantification of the discrimination index (same mice number as d). **f**, Examples trajectories from 4 mice. Two for hippocampal lesioned mice training with LEC fiber activation (top) and two normal mice training without any stimulation during training trials (bottom). Left 3 rows show trainings trials on the last training day (day4) and right 2 rows show recall trials (no food) on the next day. Red square, food zone. Grey block, landmarks (glass blocks). Error bar shows S.E.M. For all box plot, whiskers show min and max, box shows 25th, median and 75th percentile.

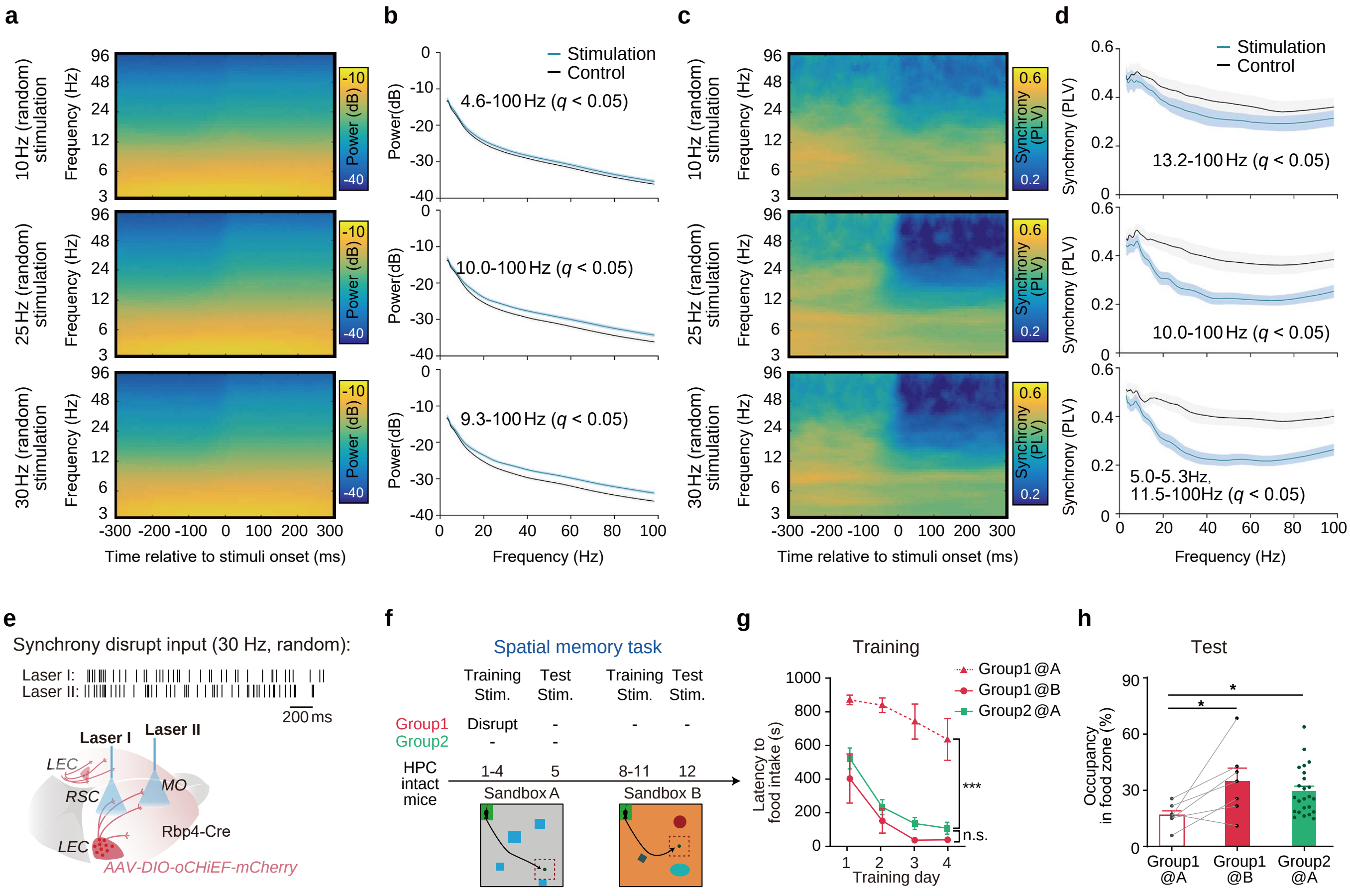

Supplementary Figure 11

Supplementary Fig. 11. Activation of LEC cortical fiber by random (asynchronous) signals results in increased LFP power and decreased synchronization.

a, Power spectrograms during LEC activation by random signals (10Hz, 25Hz, 30Hz, pulses were randomly distributive but maintain the same total pulse number in 4 seconds). **b,** Quantifications of LFP power before and after stimulation onset. Control, averaged power before stimulation. Stimulation, averaged power after stimulation. (Number of electrodes: 10Hz = 22, 25Hz = 26, 30Hz = 26, $q < 0.05$, FDR corrected, significantly changed frequency ranges is noted on the graph). Shadow of line plot shows S.E.M. **c,** Cortical synchrony spectrograms during LEC activation by random signals (10-30Hz). **d,** Quantifications of synchrony before and after stimulation onset. Control, averaged synchrony before stimulation. Stimulation, averaged synchrony after stimulation. (Number of electrode pairs: 10Hz = 11, 25Hz = 13, 30Hz = 13, $q < 0.05$, FDR corrected, significantly changed frequencies were noted on the graph). Shadow of line plot shows S.E.M. **e-h**, Asynchronous activating LEC fibers in HPC intact animals decrease spatial learning and memory recall performance. **e-f**, Experimental diagram, asynchronous input to two brain regions is to disrupt the cortical synchrony during spatial memory encoding but not during the memory test. **g,** Learning curve ($N_{Group1} = 7$; $N_{Group2} = 22$; Two-way ANOVA, Interaction: $F(6, 140) = 0.8265$, P = 0.5513, Factor: Training day $F(3, 140) = 12.45$, P < 0.0001, Factor, $F(2,140) = 69.18$, P < 0.0001, Multiple comparison: Group1@A vs. Group2@A, P < 0.0001, Group1@B vs. Group2@A, P = 0.0906). **h,** Memory test (Same mouse number as G, one-way ANOVA followed by Bonferroni's multiple comparisons to Group1@A. test. F(2, 35) = 3.8, P = 0.0312, $P_{Group1@A\ vs.\ Group1@B} = 0.0235$, $P_{Group1@A\ vs.\ Group2@A} = 0.0493$). The result of the learning curve and memory test of Group2 presented is the same as showed in Fig.3g&i, it's for comparison here. Error bar shows S.E.M.

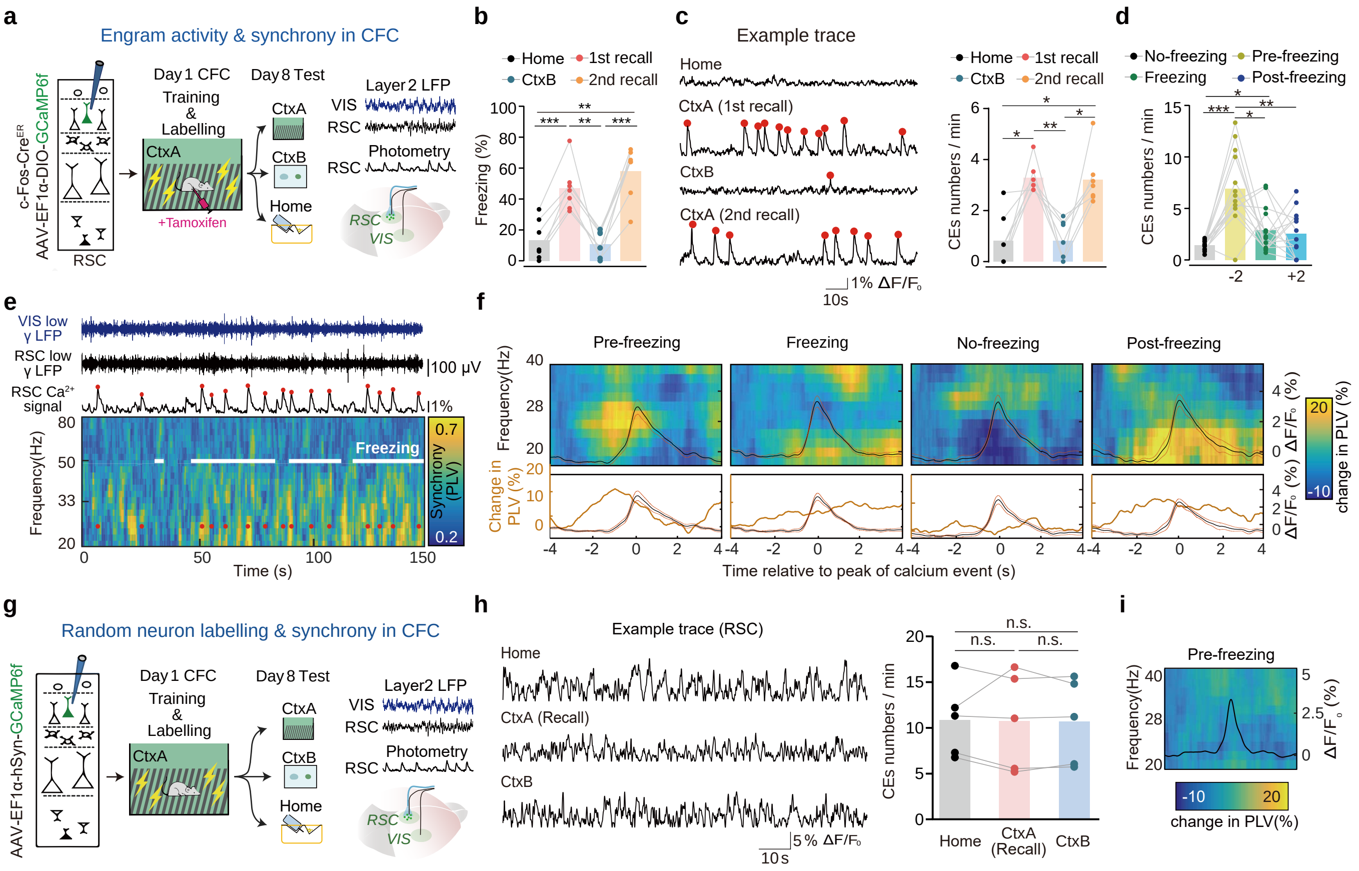

Supplementary Fig. 12. Calcium activity of labeled neurons, but not random cell populations, shows CFC memory selectivity and coupling to gamma synchrony.

a-f, Cortical theta-coupled low gamma synchrony is associated with fear memory engram activation. **a**, Experimental design of monitoring activity of cortical fear memory-related cells and the long-range gamma-synchrony simultaneously. We recorded LFP both in RSC and VIS. Activities of labeled neurons were monitored by calcium signal (GCaMP6f expressed in the activated neuron ensembles during CFC, labeled by TRAP using cFos-Cre^{ER} mice) through fiber photometry recording in RSC. **b**, Freezing levels of trained mice during memory recall (1st and 2nd recall in CtxA), homecage (Home) and exploring in a unfamiliar contextB (CtxB). (n = 7 mice, ANOVA, $F(3,18) = 31.8$, $P < 0.0001$, Tukery post-hoc test, $P_{\text{Home vs. 1st recall}} = 0.0005$, $P_{\text{Home vs. 2nd recall}} = 0.0015$, $P_{\text{1st recall vs. CtxB}} = 0.009$, $P_{\text{2nd recall vs. CtxB}} = 0.0007$). **c**, Left, an example of RSC engram calcium signals. Right, labeled RSC neurons showed context selectivity (N = 7 mice, ANOVA, $F(3,18) = 17.7$, $P < 0.0001$, Tukery post-hoc test, $P_{\text{Home vs. 1st recall}} = 0.0143$, $P_{\text{Home vs. 2nd recall}} = 0.0322$, $P_{\text{1st recall vs. CtxB}} = 0.0076$, $P_{\text{2nd recall vs. CtxB}} = 0.0206$), CEs, calcium events. **d**, CEs frequencies of different states during fear memory recall, including pre-freezing (from -3s to freezing onset), freezing (from freezing onset to end) and post-freezing (from freezing end to +3s), no-freezing (time range did not belong to any aforementioned states), both 1st and 2nd recall trials were included, n = 14 trials from 7 mice, ANOVA, $F(3,39) = 13.4$, $P < 0.0001$, Tukery post-hoc test, $P_{\text{No-freezing vs. Pre-freezing}} = 0.0002$, $P_{\text{No-freezing vs. Freezing}} = 0.0368$, $P_{\text{Freezing vs. Pre-freezing}} = 0.038$, $P_{\text{Pre-freezing vs. Post-freezing}} = 0.0032$). **e**, Top, example signals of

low gamma LFP from VIS and RSC. Bottom, calcium signal of RSC engram and low gamma synchrony (RSC-VIS) spectrogram from the same mice, demonstrating the correspondence between engram activity and cortical low gamma synchrony. Red dots, the peaks of the detected calcium events. The time course of freezing is indicated by white lines. **f**, Top, averaged low gamma synchrony (RSC-VIS) spectrogram, which was aligned to each peak (t = 0) of calcium event within each behavioral group, and normalized to baseline synchrony of each calcium event (mean PLV from -4s to -3s). The black curve inside the graph shows the averaged curve of calcium events within that phase. Bottom, change in synchrony (averaged across low gamma band) and same averaged curve of CEs are plotted for clarity of peak time of the change in synchrony. 74 CEs within the pre-freezing state, 123 CEs within the freezing state, 51 CEs within no-freezing and 55 CEs within post-freezing from all 7 animals. Dash lines plot around the CEs curve show S.D. **g-i**, GCaMP6f activities from random cell populations were not accompanied by the increase of gamma synchrony. **g**, Experimental scheme for recording the activity of randomly labeled RSC neurons and LFP during fear memory recall, same behavioral paradigm as panel a but with EF1α-hSyn-GCaMP6f to express calcium sensory in random neurons. **h**, Left, example traced of calcium signals from randomly labeled RSC neurons in different contexts. Right, quantification of calcium event frequencies (n = 5 mice, ANOVA, $F(2,8) = 0.025$, $P = 0.9757$, Tukery post-hoc test, no significance was detected). **i**, Averaged gamma synchrony (RSC-VIS) spectrogram, which was aligned to the peak of RSC calcium events during the pre-freezing phase. The black curve inside the graph shows the averaged CEs (n = 167 calcium events).

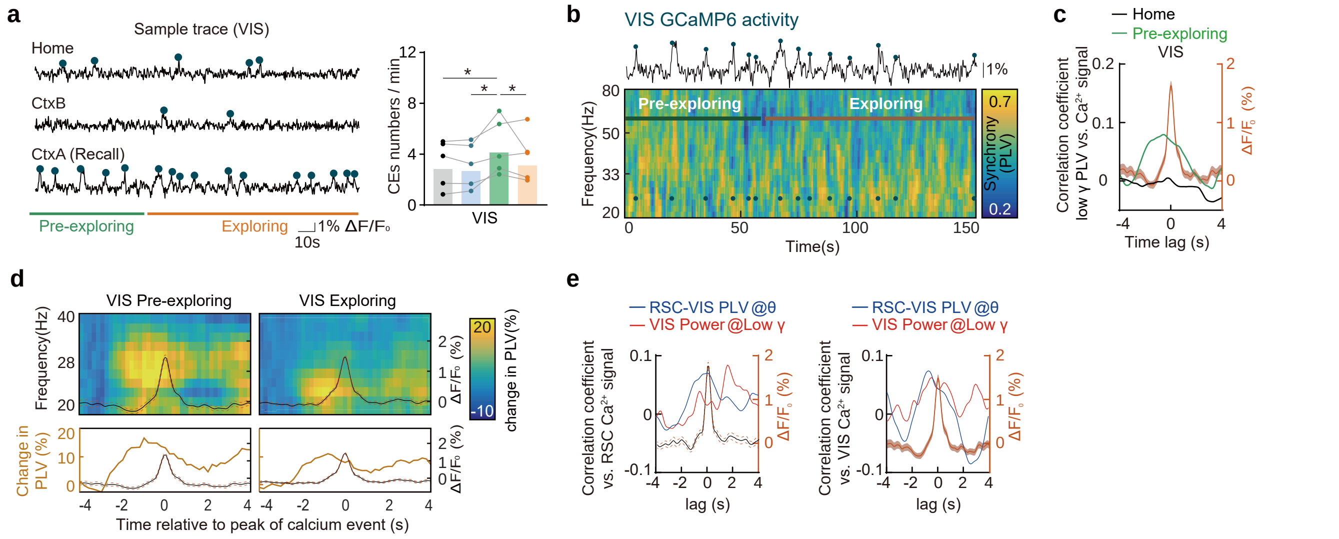

Supplementary Fig. 13. Calcium activity of labeled neurons in VIS was coupled to cortical synchrony during spatial memory recall.

a-e, Cortical synchrony was coupled to the activation of memory-related cells in VIS during spatial memory recall. **a**, Left, examples of calcium signals of VIS engram in contexts. Right, labeled VIS neurons showed context selectivity. Green dots indicate the peaks of the detected calcium events (N = 5 mice, ANOVA, $F(3, 12) = 5.1$, $P = 0.0171$, Tukery post-hoc test, $P_{\text{Pre-exploring vs. CtxB}} = 0.0301$, $P_{\text{Pre-exploring vs. Home}} = 0.0407$, $P_{\text{pre-exploring vs. Exploring}} = 0.0345$). **b**, An example of gamma synchrony spectrogram (pair of RSC-VIS) plotting together with calcium signal from VIS engram, showing the correspondence between engram activity and gamma synchrony. **c,** Cross-correlation analysis. Averaged correlation coefficient between low gamma synchrony and engram activity of VIS (right, n = 5) in pre-exploring phase (green) and homecage (black). Orange line, averaged calcium events (n = 68 VIS CEs). **d**, Averaged synchrony (RSC-VIS) spectrogram aligned to the peak of VIS calcium events during the pre-exploring and exploring phase, 68 CEs of VIS within the pre-exploring phase, and 126 CEs of VIS within exploring from 5 animals. **e**, Additional cross-correlation analysis for reference. Averaged correlation coefficient of theta synchrony (blue) and low gamma power (red) vs. calcium activity of labeled neurons in RSC (left, n = 6) or VIS (right, n = 5) in pre-exploring phase.

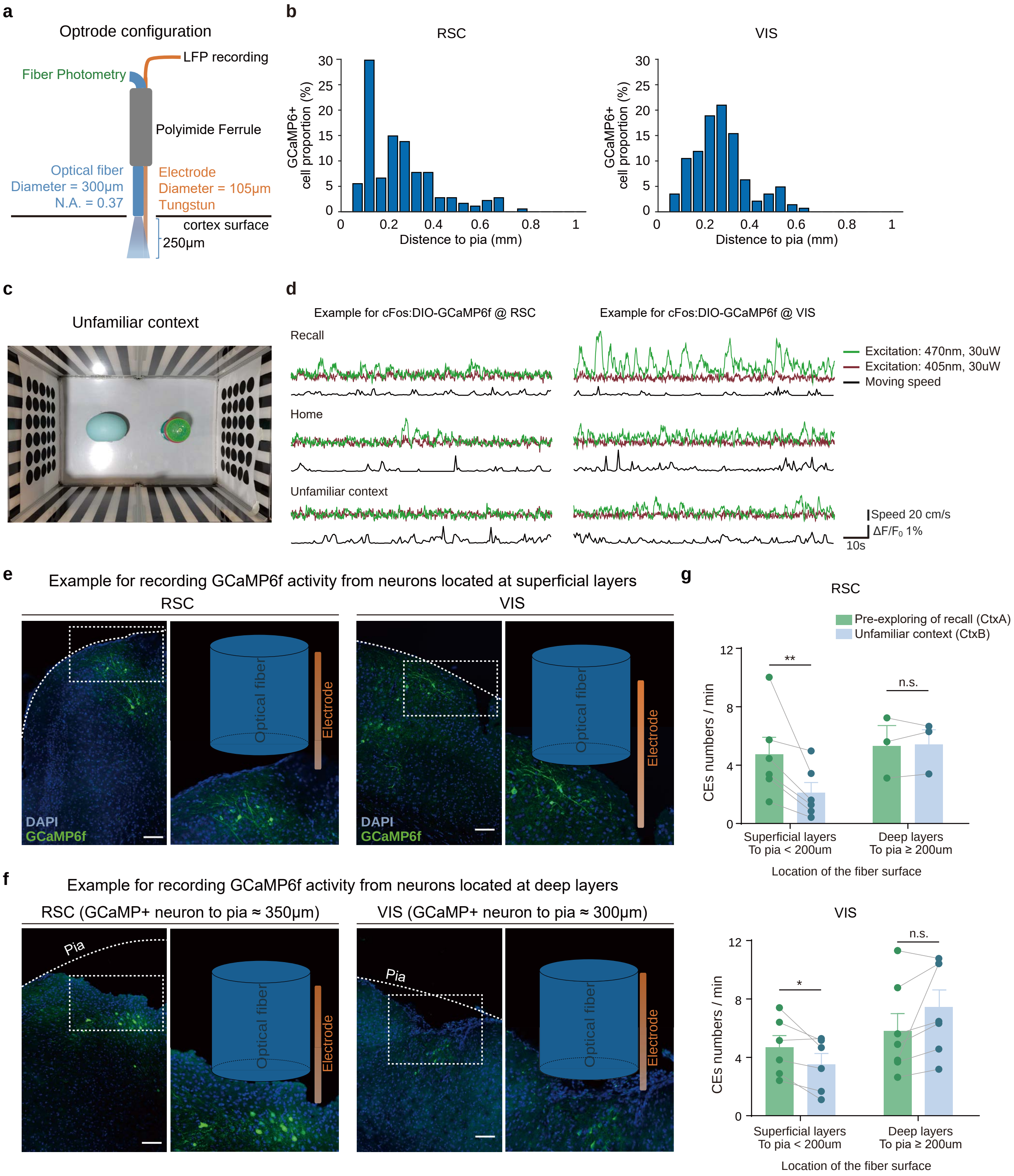

Supplementary Figure 14

Supplementary Fig. 14. Simultaneous recording calcium signals from memory-related neurons and LFP from superficial layers.

a, Illustration of custom-made optrode for simultaneous recording of LFP and calcium signal. Optrodes were implanted above the virus injection sites and sealed by black dental cement. Noted that the tip of optical fiber was placed on the surface of the cortex (DV = -0.0mm) and the protruding electrode was implanted into the cortex with the depth 0.25 mm. **b,** The distribution of labeled neurons in RSC and VIS. RSC (111 cells from 7 mice. VIS, 134 cells from 7 mice). **c,** Picture of contextB used for fear conditioning and spatial memory task as a unfamiliar context. **d,** Example signals from GCaMP6 of labeled neurons in RSC (left) and VIS (right), exiting by 470 nm and 405 nm light simultaneously. Green, fluorescence (470 nm excitation), red (405 nm, excitation), Black, moving speed. **e,** Example of recording GCaMP6f activities from neurons located at superficial layers of RSC and VIS. Confocal images in the left panel together with its enlarged images of the white box area in the right panel confirm the implantation location of the optrode. Scale bar, 100 μm. **f,** Example of recording GCaMP6f activities from neurons located at deep layers. Scale bar, 100 μm. **g,** Quantification of calcium event frequencies in pre-exploring and in the unfamiliar context of two groups of mice with different optrode recording depth (The superficial group in RSC or VIS is the same as Fig. 5b and supplementary Fig.13a; For deep cortex group of RSC, n = 3, Pre-Exploring vs. CtxB, two-sided paired *t*-test, $t(2) = 0.64$, $P = 0.5866$, for deep cortex group of VIS, n = 7, Pre-Exploring vs. CtxB, two-sided paired *t*-test, $t(6) = 2.2$, $P = 0.073$). Error bar shows S.E.M.

a

Mouse ID	Brain-Region	At least one object tuning >= 95%	Preferred distance (cm)							Angle (rad)						
			Wall	Food zone	Start block	Block 2	Block 3	Block 4	Block 5	Wall	Food zone	Start block	Block 2	Block 3	Block 4	5
16#	RSC	Yes	21.5	17.5	12.4	26.2	2.4	3.0	21.7	-0.5	-3.0	2.7	1.6	-2.4	-1.2	2.7
18#	RSC	Yes	27.0	24.9	26.4	18.3	5.5	6.8	20.3	1.4	1.5	1.1	0.8	2.0	2.1	1.1
20#	RSC	No	12.9	22.6	26.0	17.6	9.6	8.3	13.1	0.2	0.0	1.4	-2.9	-1.6	-1.0	3.0
22#	RSC	No	13.3	16.3	17.9	13.9	28.2	21.5	8.0	-3.1	-0.8	-1.8	0.4	0.9	1.5	-0.4
27#	RSC	Yes	15.7	21.1	19.1	17.9	4.2	4.4	13.5	0.4	-2.5	0.1	-0.7	0.9	2.4	-2.3
39#	VIS	Yes	24.4	23.6	13.2	18.8	24.6	21.5	4.4	2.2	-2.9	1.0	-2.3	-1.8	-1.2	-3.0
40#	VIS	No	11.4	26.8	17.9	12.2	8.7	21.3	22.2	0.5	1.2	-0.8	-2.8	1.5	2.2	1.0
41#	VIS	Yes	23.2	3.3	5.2	20.1	16.4	21.1	24.2	-2.0	0.2	0.2	2.2	1.3	-1.6	-0.7
42#	VIS	Yes	28.0	20.0	17.3	20.5	22.5	15.3	21.9	-0.9	0.2	2.4	-0.2	1.5	2.1	-0.5
44#	RSC	No	16.4	27.8	17.6	17.2	6.5	11.8	21.8	3.1	2.3	-1.7	-0.3	1.1	1.7	-2.2
45#	VIS	Yes	25.3	21.0	21.2	5.8	3.2	17.9	24.7	2.5	1.9	-1.9	-1.2	-3.0	-2.0	2.0
46#	RSC	Yes	21.1	22.1	8.6	8.4	26.7	26.0	23.9	-1.2	0.5	2.9	1.6	1.2	2.0	1.9
48#	RSC	No	26.3	26.6	17.2	18.2	10.5	17.7	7.0	-0.5	0.8	-1.9	0.4	1.0	-0.2	-0.1

b

Mouse ID	Brain-Region	Allocentric Map Occupancy (%)	Egocentric map occupancy (%)						
			Wall	Food zone	Start block	Block 2	Block 3	Block 4	Block 5
16#	RSC	72.32	100.0	100.0	97.5	99.7	99.9	100.0	100.0
18#	RSC	66.24	100.0	100.0	94.9	99.9	99.8	97.2	99.5
20#	RSC	56.64	99.9	99.9	80.9	97.1	99.2	90.5	99.6
22#	RSC	82.72	100.0	100.0	100.0	99.9	100.0	98.9	100.0
27#	RSC	61.12	100.0	99.9	95.4	100.0	97.8	91.6	100.0
39#	VIS	87.36	100.0	100.0	99.7	100.0	100.0	100.0	100.0
40#	VIS	86.4	100.0	100.0	99.7	98.2	99.9	99.1	100.0
41#	VIS	80.8	99.9	100.0	100.0	99.9	98.8	100.0	100.0
42#	VIS	75.84	100.0	100.0	96.2	99.9	98.9	97.9	99.9
44#	RSC	82.72	100.0	100.0	99.8	100.0	100.0	99.6	100.0
45#	VIS	68.96	100.0	100.0	98.2	99.7	99.7	97.4	98.9
46#	RSC	70.56	100.0	99.4	98.5	99.2	99.9	93.5	99.8
48#	RSC	64.8	100.0	100.0	93.9	98.8	100.0	94.9	99.8
Average	/	73.6	100.0	99.9	96.5	99.4	99.5	97.0	99.8

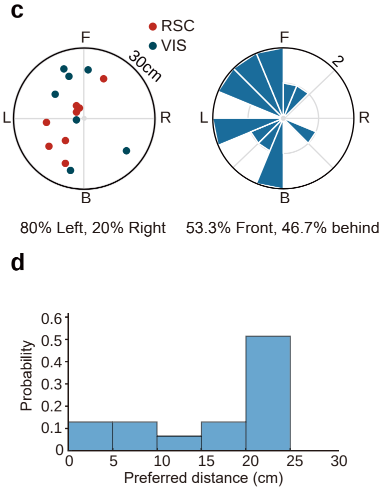

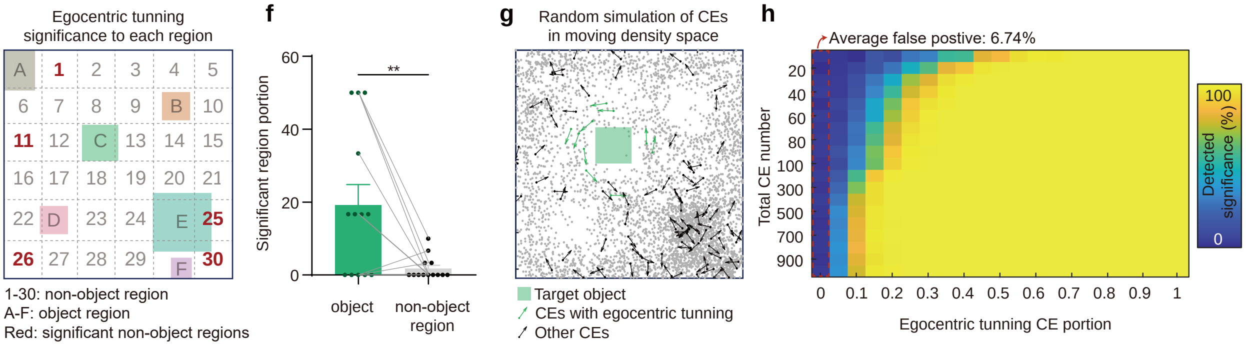

Supplementary Figure 15

Supplementary Fig. 15. Properties about egocentric coding of labeled population.

a, A summary table shows the preferred distance and angle of receptive fields of each EOR for all tuning from 13 mice. 15 EORs show significance (green background in the table). **b**, Allocentric map occupancy and egocentric map occupancy. Green-filled square noted the significant EOR coding. **c,** Left, Polar scatter plot of preferred orientation versus preferred distance for all significant EORs. Right, Polar histogram of preferred receptive field orientations for all significant EORs. **d,** Distribution of preferred distance of all significant EORs receptive fields. **e-f,** Activities of labeled neurons are more likely to show significant egocentric coding to objects than to non-object regions. **e**, The definition of the object position (A-E) and non-object regions (1-30), red numbers indicates all the regions detected as significant. **f**, The comparison of the significant portion between objects and non-object regions. Significant objects portion = 100% * (a/6); Significant non-region portion = 100% * (b/30), here a represent the number of significant egocentric coding to objects and b represent the number of significant egocentric coding to non-object regions (two-sided paired t-test, n = 13, $P = 0.0075$, $t(12) = 3.2$). Error bar shows S.E.M. **g-h,** Simulation to test whether the egocentric coding significance detection is reliable. **g,** A simulation example for one simulation. Mouse position was generated according to real recall occupancy map (Fig. 3h), the head direction was random from -π to-π radian. Points with calcium event (CEs) include a portion of egocentric tuning CEs to a virtual object (green square and surround green points) and other CEs (black), corresponding head directions were marked by arrows, in total 4200 points (14min * 60 sec * 5 fps). **h,** Simulation was repeated 500 times for each pair between total CE number and egocentric tuning CE portion to calculate the rate of detected significance.

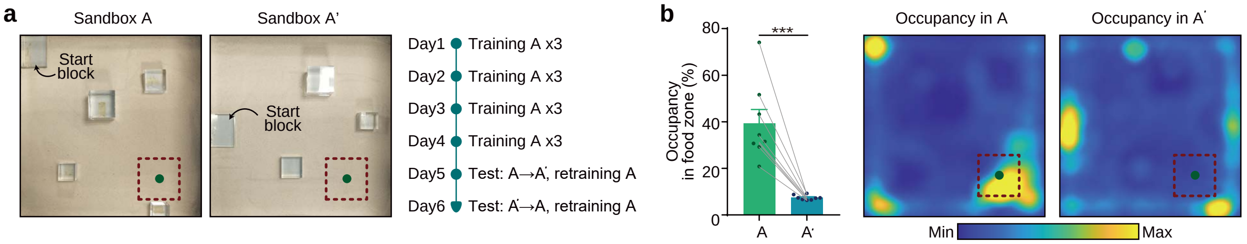

Supplementary Fig. 16. Landmark relocation in sandbox largely impairs performance in the spatial memory task.

a, Left, Two images show the original sandbox (A) and the changed object sandbox (A'). Right, behavioral paradigm. **b,** Left, occupancy in foodzone. (N = 8 mice, two-sided paired t-test, $t(7) = 5.7$, $P = 0.0008$). Error bar shows S.E.M. Right, the average occupancy map of recall in A and in A'.

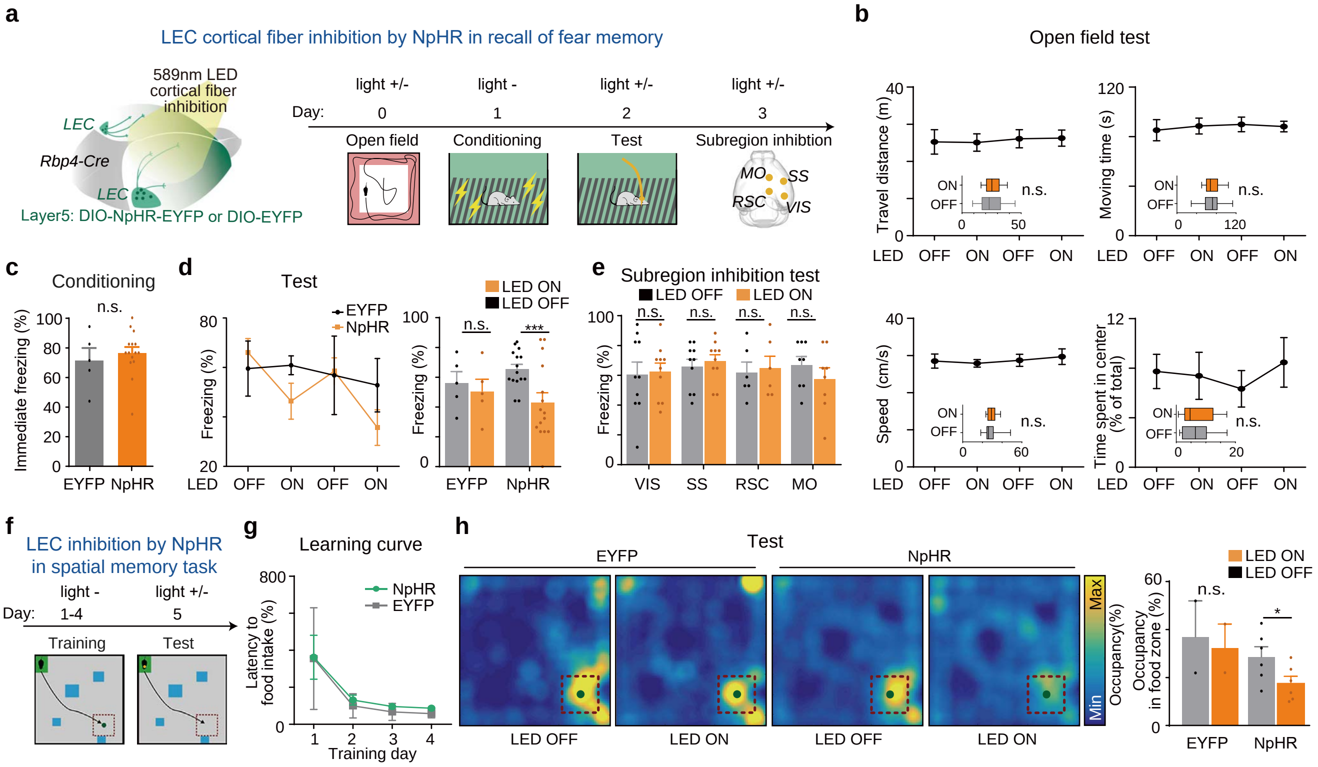

Supplementary Figure 17

Supplementary Fig. 17. Inhibition of cortical LEC fibers impairs memory retrieval in the contextual fear conditioning task and spatial memory task.

a, Illustration of inhibiting LEC fibers during fear conditioning. Left, NpHR were specifically expressed in layer5 neurons by using Rbp4-Cre mice. 589 nm LED light through the carinal window was used to achieve inhibition of LEC cortical fibers. The right panel shows the behavior paradigm. First, mice underwent an open field test for 12 mins (OFF-ON-OFF-ON, 3 mins for each block) to test the behavioral side effects of LEC cortical fibers inhibition. Next, two groups of mice received fear conditioning, followed by a recall test trial with or without light 24 hours later. Finally, mice went through a subregion inhibition test (only inhibit one of the four subregions in recall trials and keep other brain regions unaffected). **b,** Quantifications of four parameters of open field test (n = 14 mice), including travel distance (P = 0.5163), moving speed (P = 0.3209), moving time (P = 0. 9942) and time spent in center (P = 0.3856). All statistical significance was assessed by two-sided paired *t*-test. **c,** Immediate freezing levels during conditioning (EYFP: n = 5; NPHR: n = 15; two-sided unpaired *t*-test, P = 0.5905). **d,** Inhibition of LEC cortical fiber leads to impairment of fear memory recall. Left, LED OFF-ON-OFF-ON test (EYFP: n = 5; NpHR, n = 15). Right, quantification (EYFP OFF vs. ON, two-sided paired *t*-test, P = 0.4954, NpHR OFF vs. ON, two-sided paired *t*-test, $P < 0.0001$). **e,** Subregion inhibition test (inhibition area = 0.5 mm^2, VIS: n = 10, two-sided paired *t*-test: P = 0.8521, SS: n = 10, two-sided paired *t*-test: P = 0.6075, RSC: n = 6, paired *t*-test: P = 0.5177, MO: n = 8, two-sided paired *t*-test: P = 0.2209). **f,** Spatial memory task paradigm. **g,** Learning curve of two groups. (EYFP: n = 2; NpHR: n = 6). **h,** Left, occupancy map of memory test. Right, quantification of occupancy in food zone (Two-way ANOVA followed by Bonferroni's multiple comparisons, $F_{\text{EYFP vs. NpHR}}$ (1,6) = 1.8, P = 0.2314, $F_{\text{ON vs OFF}}$ (1,6) = 9.3, P = 0.0226, $F_{\text{Interaction}}$ (1,6) = 1.5, P = 0.2689, $P_{\text{EYFP ON vs. EYFP OFF}}$ = 0.6632; $P_{\text{NpHR ON vs. NpHR OFF}}$ = 0.0106). n.s., no significance. *P <0.05, ***P < 0.0001. Error bars show mean ± S.E.M.

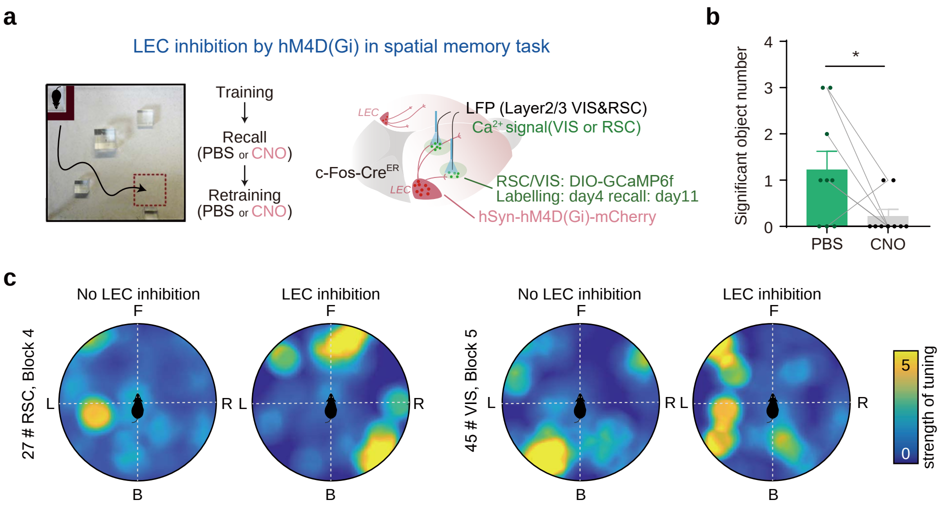

Supplementary Fig. 18. LEC inhibition distorts egomaps.

a, Experimental diagram for LEC inhibition during memory recall in the spatial memory task. Same dataset as figure 6. **b,** Number of the significant egocentric coding object with or without LEC inhibition, CNO dosage: 2 mg/kg. Same mice as in figure 7, (N = 8, two-sided paired t-test, $t(7) = 3.4$, $P = 0.0112$). **c,** Two examples of egomap of the same object with or without LEC inhibition to show distortion. $^*P < 0.05$, Error bar shows S.E.M.

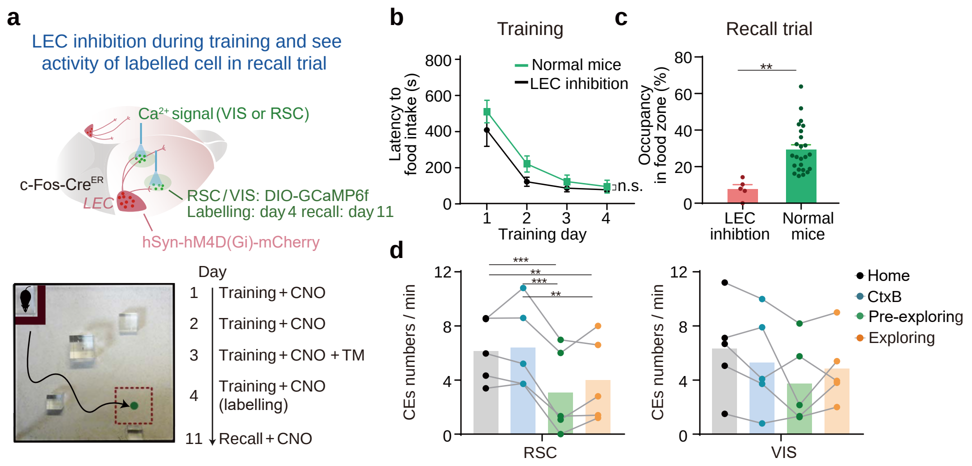

Supplementary Fig 19. The absence of LEC impairs memory formation and disrupts the normal firing of engram neurons.

a, Experimental design. LEC was infected by hSyn-hM4D(Gi)-mCherry, CNO dosage: 2mg/kg. **b**, Learning curves. (LEC inhibition: N = 5, Normal mice group = 24, two-way ANOVA, Group factor: $F(1, 27) = 0.9259$, $P = 0.3445$; Time factor: $F(3, 81) = 22.91$, $P < 0.0001$; Interaction: $F(3, 81) = 0.3704$, $P = 0.7746$, Bonferroni's post-hoc test, $P_{Day1} = 0.9976$, $P_{Day2} > 0.9999$, $P_{Day3} > 0.9999$, $P_{Day4} > 0.9999$). **c**, Quantification of occupancy in food zone. (same mice as a, two-sided paired t-test, $t(27) = 3.651$, $P = 0.0011$). The result of the learning curve and memory test of normal mice group presented is the same as shown in Fig. 3g&i, it's for comparison here. The mice seemed to know in general that there was food in the scene, but not exactly where. This illustrates the importance of LEC for accurate memory formation. **d**, CEs frequency in day11. Comparing with context-selective RSC and VIS activity in LEC intact mice (Fig. 5B), neither labeled RSC nor VIS neurons showed context selectivity in LEC inhibition mice (N_{RSC}= 5; one-way ANOVA, $F(3, 12) = 19.8$, $P < 0.001$. Tukery post-hoc test, $P_{\text{Home vs. Pre-exploring}} = 0.0004$, $P_{\text{Home vs. Exploring}} = 0.0064$, $P_{\text{CtxB vs. Pre-exploring}} = 0.0002$, $P_{\text{CtxB vs. Exploring}} = 0.0027$. N_{VIS}= 5; one-way ANOVA, $F(3, 12) = 2.5$, $P = 0.1051$).
