# Supplementary material for: Acquiring new memories in neocortex of hippocampal-lesioned mice
Source: Nat Commun. 2022 Mar 24;13:1601. doi: 10.1038/s41467-022-29208-5 (PMC8948206; doi:10.1038/s41467-022-29208-5)
Supplement: Supplementary file 2 — Reporting Summary [file 41467_2022_29208_MOESM2_ESM.pdf]

nature portfolio

Corresponding author(s): Ji-song Guan

Last updated by author(s): Feb 11, 2022

Reporting Summary

Nature Portfolio wishes to improve the reproducibility of the work that we publish. This form provides structure for consistency and transparency in reporting. For further information on Nature Portfolio policies, see our Editorial Policies and the Editorial Policy Checklist.

Statistics

For all statistical analyses, confirm that the following items are present in the figure legend, table legend, main text, or Methods section.

n/a	Confirmed	
☐	☒	The exact sample size (n) for each experimental group/condition, given as a discrete number and unit of measurement
☐	☒	A statement on whether measurements were taken from distinct samples or whether the same sample was measured repeatedly
☐	☒	The statistical test(s) used AND whether they are one- or two-sided<br>*Only common tests should be described solely by name; describe more complex techniques in the Methods section.*
☒	☐	A description of all covariates tested
☐	☒	A description of any assumptions or corrections, such as tests of normality and adjustment for multiple comparisons
☒	☐	A full description of the statistical parameters including central tendency (e.g. means) or other basic estimates (e.g. regression coefficient) AND variation (e.g. standard deviation) or associated estimates of uncertainty (e.g. confidence intervals)
☐	☒	For null hypothesis testing, the test statistic (e.g. F, t, r) with confidence intervals, effect sizes, degrees of freedom and P value noted<br>*Give P values as exact values whenever suitable.*
☒	☐	For Bayesian analysis, information on the choice of priors and Markov chain Monte Carlo settings
☒	☐	For hierarchical and complex designs, identification of the appropriate level for tests and full reporting of outcomes
☐	☒	Estimates of effect sizes (e.g. Cohen's d, Pearson's r), indicating how they were calculated

Our web collection on statistics for biologists contains articles on many of the points above.

Software and code

Policy information about availability of computer code

Data collection	Apollo 1.0.0 was applied in LFP collection.<br>Brain slices were imaged using a ZEISS (LSM710META) confocal microscope. ImageJ v1.52p was applied in imaging analysis.<br>The fiber photometry system was bought from Thinker Tech Nanjing Biotech Limited Co.<br>Skeleton analysis is from Matlab R2020b (MathWorks) bwmorph function and was applied in determining head directions of mice.
Data analysis	All analyses were performed using custom code written in MATLAB R2020b (MathWorks). In addition, as mentioned below, some analyses used functions available in open-source MATLAB code:<br>1. The Torrence & Compo wavelet(v1.0): http://paos.colorado.edu/research/wavelets/<br>2. Chronux toolboxes(2.12v03): http://chronux.org/<br>GraphPad Prism version 7.00 (GraphPad Software, La Jolla, California, USA) and OriginPro version 9.0 (OriginLab Software, Northampton, Massachusetts, USA) were used for statistical analyses.<br>Custom Matlab code supporting this study will be available at https://figshare.com. DOI:10.6084/m9.figshare.17490371

For manuscripts utilizing custom algorithms or software that are central to the research but not yet described in published literature, software must be made available to editors and reviewers. We strongly encourage code deposition in a community repository (e.g. GitHub). See the Nature Portfolio guidelines for submitting code & software for further information.

Data

Policy information about availability of data

All manuscripts must include a data availability statement. This statement should provide the following information, where applicable:

- Accession codes, unique identifiers, or web links for publicly available datasets
- A description of any restrictions on data availability
- For clinical datasets or third party data, please ensure that the statement adheres to our policy

Source data are available at https://figshare.com. DOI:10.6084/m9.figshare.17644445. All data supporting the findings of this study are available from the corresponding author upon reasonable request.

Field-specific reporting

Please select the one below that is the best fit for your research. If you are not sure, read the appropriate sections before making your selection.

[x] Life sciences   [ ] Behavioural & social sciences   [ ] Ecological, evolutionary & environmental sciences

For a reference copy of the document with all sections, see nature.com/documents/nr-reporting-summary-flat.pdf

Life sciences study design

All studies must disclose on these points even when the disclosure is negative.

Sample size	Sample size were determined based on established practice and on our previous experience in respective assays [Ding et al.,2017; Xie at al., 2014;].
Data exclusions	Position of virus injection sites, hippocampus lesion sites or electrode / optrode implantation sites were confirmed by brain slice imaging after behavioral experiments . Mice with wrong positions (mentioned above) were excluded.<br>LFP data with bad electrical grounding or strong 50Hz electromagnetic noise were excluded.
Replication	Experiments in this study were all replicated and reproduced. N numbers for each experiment were mentioned in figure legends.
Randomization	Mice were age-matched and randomized where appropriate (e.g. prior to stereotaxic surgery).<br>The experimental sequencing and surrogate PLV calculation were randomized by MATLAB rand function to produced random sequence.
Blinding	Freezing behavior, defined as a lack of movement except for heart beat and respiration associated with a crouching posture, was recorded by video and rated by two blinded observers (unaware of the experimental conditions) during 3 min.<br>Blinding was performed in the hippocampus lesion experiments, the experimenter didn't know which group of mice were hippocampus lesioned.<br>No blinding was performed in the in vivo recording experiments due to requirements for cage labeling.

Reporting for specific materials, systems and methods

We require information from authors about some types of materials, experimental systems and methods used in many studies. Here, indicate whether each material, system or method listed is relevant to your study. If you are not sure if a list item applies to your research, read the appropriate section before selecting a response.

Materials & experimental systems

n/a	Involved in the study	
x		Antibodies
x		Eukaryotic cell lines
x		Palaeontology and archaeology
	x	Animals and other organisms
x		Human research participants
x		Clinical data
x		Dual use research of concern

Methods

n/a	Involved in the study	
x		ChIP-seq
x		Flow cytometry
x		MRI-based neuroimaging

Animals and other organisms

Policy information about studies involving animals; ARRIVE guidelines recommended for reporting animal research

Laboratory animals	Wild type (C57BL/6) and Ai9 (007909, The Jackson Laboratory) male mice aged 3–5 months were used. c-FOS-CreERT2 mice were obtained from Jackson Laboratories,adult male mice aged 3 months were used. Tg (Rbp4-Cre) KL100Gsat/Mmcd (denoted as Rbp4-

	Cre, GENSAT RP24-285K21), adult male mice aged 3 months were used. The mice were housed in homecage in a humidity- and temperature-controlled environment.
Wild animals	This study did not involve wild animals.
Field-collected samples	This study did not involve samples collected from the field.
Ethics oversight	Animal care was in accordance with the Institutional Guidelines of Tsinghua University. The Laboratory Animal Facility at the Tsinghua University is accredited by Association for Assessment and Accreditation of Laboratory Animal Care International (AAALAC), and protocols are proved by the Institutional Animal Care and Use Committee (IACUC).

Note that full information on the approval of the study protocol must also be provided in the manuscript.
