# Supplementary material for: Acquiring new memories in neocortex of hippocampal-lesioned mice
Source: Nat Commun. 2022 Mar 24;13:1601. doi: 10.1038/s41467-022-29208-5 (PMC8948206; doi:10.1038/s41467-022-29208-5)
Supplement: Supplementary file 4 — Description of Additional Supplementary Files [file 41467_2022_29208_MOESM4_ESM.pdf]

Title: Supplementary Movie 1.
Description: Generation of egocentric maps. The red square represents the biggest block, the blue square represents a nonobject region. The green square represents the food zone. The video is made from a 3.5 minutes recall trial.
